# Laparoscopic resection of a giant post-mesenteric ovarian seromucinous cystadenoma: a case report and review

**DOI:** 10.3389/fonc.2025.1576522

**Published:** 2025-06-12

**Authors:** Hong Chen, Yifan Ye, He Liu, Rui Huang, Ningbo Li, Yaling Tang

**Affiliations:** ^1^ Department of Obstetrics and Gynecology, The First Affiliated Hospital of Xiamen University, Xiamen, China; ^2^ Department of Clinical Medicine, School of Medicine, Fujian Medical University, Fuzhou, China; ^3^ Department of Gastrointestinal Surgery, The First Affiliated Hospital of Chongqing Medical University, Chongqing, China; ^4^ Department of Pathology, The First Affiliated Hospital of Xiamen University, Xiamen, China

**Keywords:** laparoscopic treatment, ovarian seromucinous cystadenoma, giant cyst, post-mesenteric, single-port laparoscopic surgery

## Abstract

**Background:**

Ovarian seromucinous tumors represent a rare subclass of ovarian neoplasms. While the majority of these tumors are benign, the potential for malignant transformation persists and should be considered. To enhance patient outcome and mitigate the risk, early detection and timely intervention are paramount. In cases involving large or complex ovarian masses, open surgery is often the preferred approach, as it provides superior access for comprehensive tumor resection and enables immediate histopathological evaluation. Nevertheless, with advancements in laparoscopic techniques, single-port laparoscopic surgery has emerged as a viable alternative for patients. This approach not only demonstrates comparable effectiveness but also offers the benefits of expedited recovery and reduced scarring.

**Case report:**

A 65-year-old female presented with a seven-month history of abdominal distension, a sensation of fullness beneath the xiphoid, left-sided discomfort, and intermittent morning cramping. Laboratory findings revealed a mild elevation in CA-125 to 46 U/mL, and CT imaging suggested a diagnosis of an ovarian cystadenoma or possibly a retroperitoneal mass. Preoperative assessment was challenging due to the tumor’s irregular morphology, substantial size, and its adhesions to surrounding pelvic and abdominal structures, making it difficult to precisely determine its origin. In light of these complexities, a single-port laparoscopic approach was chosen to minimize trauma, allow for more precise handling of the tumor, and reduce the risk of cystic fluid leakage or inadvertent dissemination of the tumor. Postoperative pathological examination confirmed the lesion to be a seromucinous ovarian cystadenoma.

**Conclusion:**

This case exemplifies the imperative for a multidisciplinary approach in the diagnosis and treatment of ovarian seromucinous tumors, emphasizing the advantages of minimally invasive surgical techniques. Given the rarity of such tumors, it is essential that ongoing research into the pathogenesis, classification, and treatment strategies be prioritized to enhance patient outcomes.

## Background

Ovarian seromucinous tumors represent a rare subtype of ovarian neoplasms, having been first characterized in 2002. These tumors are distinguished by a histological architecture that incorporates both serous and mucinous (cervical-type) epithelial components. Furthermore, they may exhibit additional epithelial types, including transitional, squamous, clear cell, or endometrioid epithelium (1, 2). In certain instances, these tumors are associated with the presence of endometriosis (3–5). The 2014 revision of the *World Health Organization (WHO) Classification of Tumors of Female Reproductive Organs* (4th edition) introduced notable updates to the classification of ovarian tumors, particularly in the category of epithelial tumors. This includes an update to the grading system for serous carcinoma and the introduction of a new morphological category, the seromucinous tumor (6). Like other ovarian epithelial tumors, ovarian seromucinous tumors can be categorized into three distinct types: benign, borderline, and invasive carcinoma (6, 7). However, the current corpus of research addressing the biological characteristics, clinical progression, and standardized treatment modalities for this tumor is significantly insufficient, particularly concerning surgical management. Furthermore, there exists a conspicuous paucity of literature investigating the role of laparoscopy within this context.

In this case, preoperative imaging revealed the presence of cystic lesions, accompanied by a modest elevation in CA-125 levels. Given the tumor’s irregular shape, large size, and adhesions to surrounding structures, surgical intervention was deemed necessary. After thorough discussions with the patient and family, we detailed the risks and benefits of the available approaches. Considering the patient’s concern about abdominal scarring, we agreed to proceed with initial laparoscopic exploration while emphasizing that conversion to open surgery would occur immediately if intraoperative findings suggested malignancy. A single-port laparoscopic technique was selected to minimize tissue trauma and mitigate risks of cystic fluid leakage or tumor dissemination. Final pathology confirmed a seromucinous ovarian cystadenoma with no definitive evidence of malignancy. Nevertheless, given the potential risks associated with such tumors, ongoing monitoring and follow-up are essential to ensure long-term patient safety.

## Case presentation

**Figure 1:**
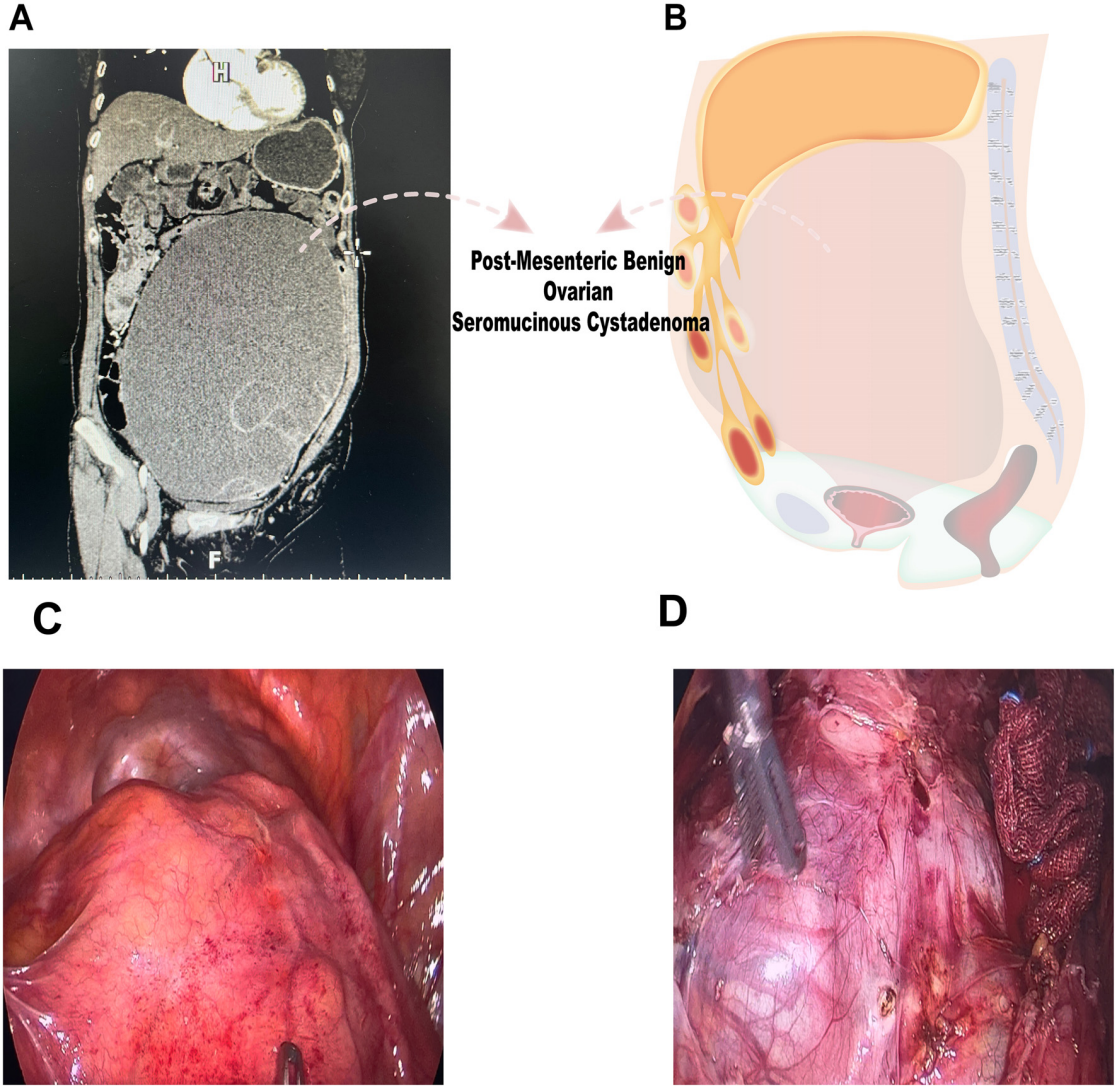
Preoperative imaging examination and intraoperative findings. **(A, B)** CT imaging demonstrated a substantial lesion within the pelvic cavity, measuring approximately 22.8 cm × 12.4 cm × 30 cm, suggesting a complex internal structure. **(C, D)** Laparoscopic examination revealed a large cystic mass with high tension, smooth surface, and clear fluid.

A 65-year-old female presented with a seven-month history of progressive abdominal distention, accompanied by epigastric fullness, left-sided discomfort, and intermittent morning cramping. An ultrasound revealed a cystic mass in the pelvic and abdominal regions, measuring approximately 32 cm×33 cm, with well-defined borders and low echogenicity. There were no significant blood flow signals detected in the surrounding tissues. During physical examination, significant abdominal distention and a thin abdominal wall were noted, with the mass’s upper edge located just below the xiphoid process. The tumor marker CA-125 was noted to be mildly elevated at 46.0 U/mL, while all other tumor markers remained within normal parameters. A gastrointestinal endoscopy successfully ruled out any digestive tract-related pathologies. Additional assessment through contrast-enhanced CT imaging revealed a sizable lesion in the pelvic cavity, measuring approximately 22.8 cm×12.4 cm×30 cm, displaying internal separation echoes (**Figures 1A, B**). Upon contrast-enhanced CT examination, the solid portions and septal regions exhibited enhancement, while the cystic areas remained non-enhancing.

A comprehensive analysis of clinical data necessitated a differential diagnosis encompassing a range of conditions, with particular emphasis on benign ovarian tumors or borderline tumors. While the potential for malignant neoplasms, such as cystadenocarcinoma, remained relatively low—bolstered by the absence of significant tumor markers or signs of metastasis. The CT scans indicated the presence of a cystic tumor, likely of serous or mucinous origin. Furthermore, it was prudent to consider several rare preoperative differential diagnoses, including retroperitoneal lymphangiomas and mesenteric tumors. Given that endoscopic examination had effectively ruled out a gastrointestinal source, the final diagnosis ultimately relied on histopathological evaluation. Based on the clinical and imaging characteristics presented, the likelihood of encountering a low malignant potential ovarian tumor appeared to be substantial. Consequently, further surgical evaluation was imperative to elucidate the nature of the mass. After a comprehensive discussion regarding the patient’s condition and thorough evaluation of any potential contraindications, a single-port laparoscopic exploration was performed. This minimally invasive approach was selected to reduce the likelihood of necessitating conversion to an open abdominal incision in the event of surgical challenges. A 2 cm incision was made at the umbilicus, followed by meticulous layer-by-layer dissection to access the abdominal cavity. Subsequently, a 60mm single-use incision fixation retractor was utilized to ensure optimal visualization and accessibility throughout the procedure.

**Figure 2:**
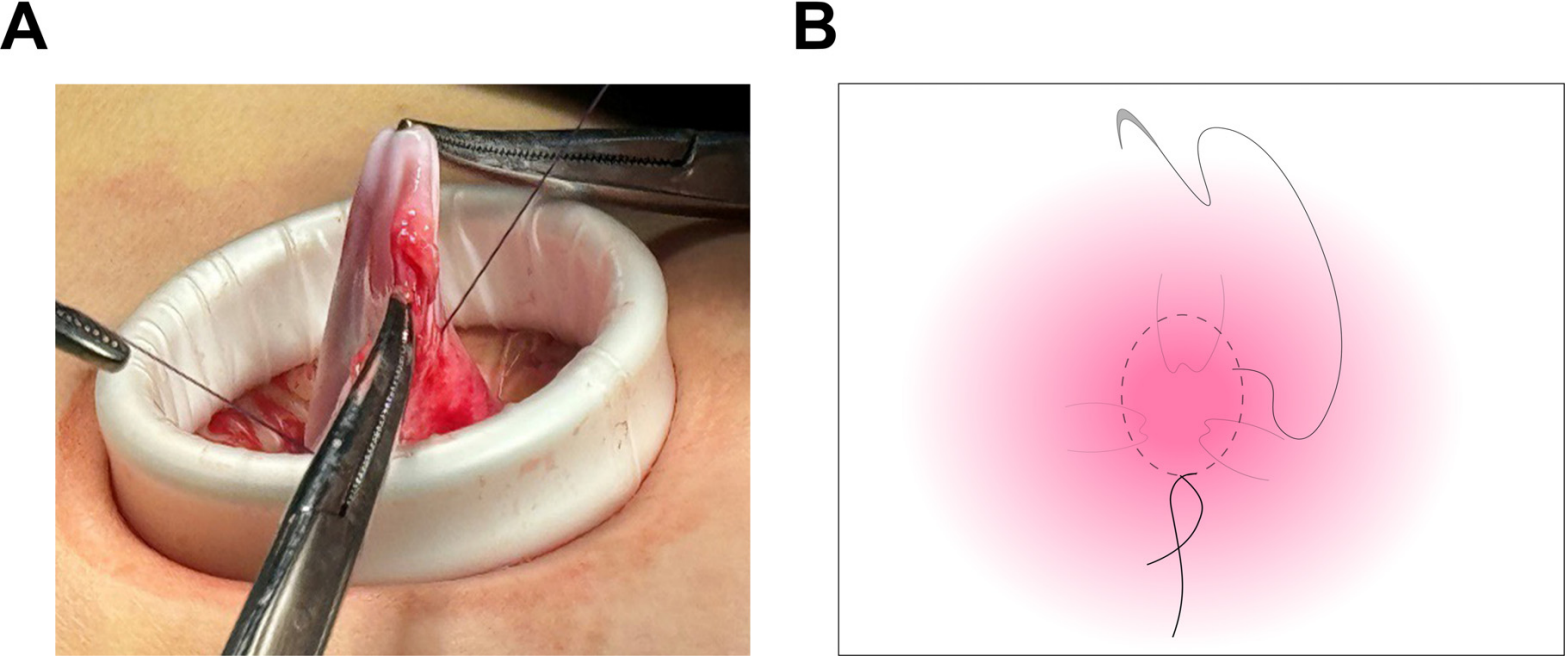
Suturing of the cyst wall using a purse-string technique. **(A)** The cyst wall was meticulously sutured utilizing a purse-string technique, ensuring the outlet was securely sealed. This approach effectively prevented fluid leakage and minimized the risk of spillage throughout the procedure. **(B)** The diagram demonstrates the suturing of the cyst wall.

**Figure 3:**
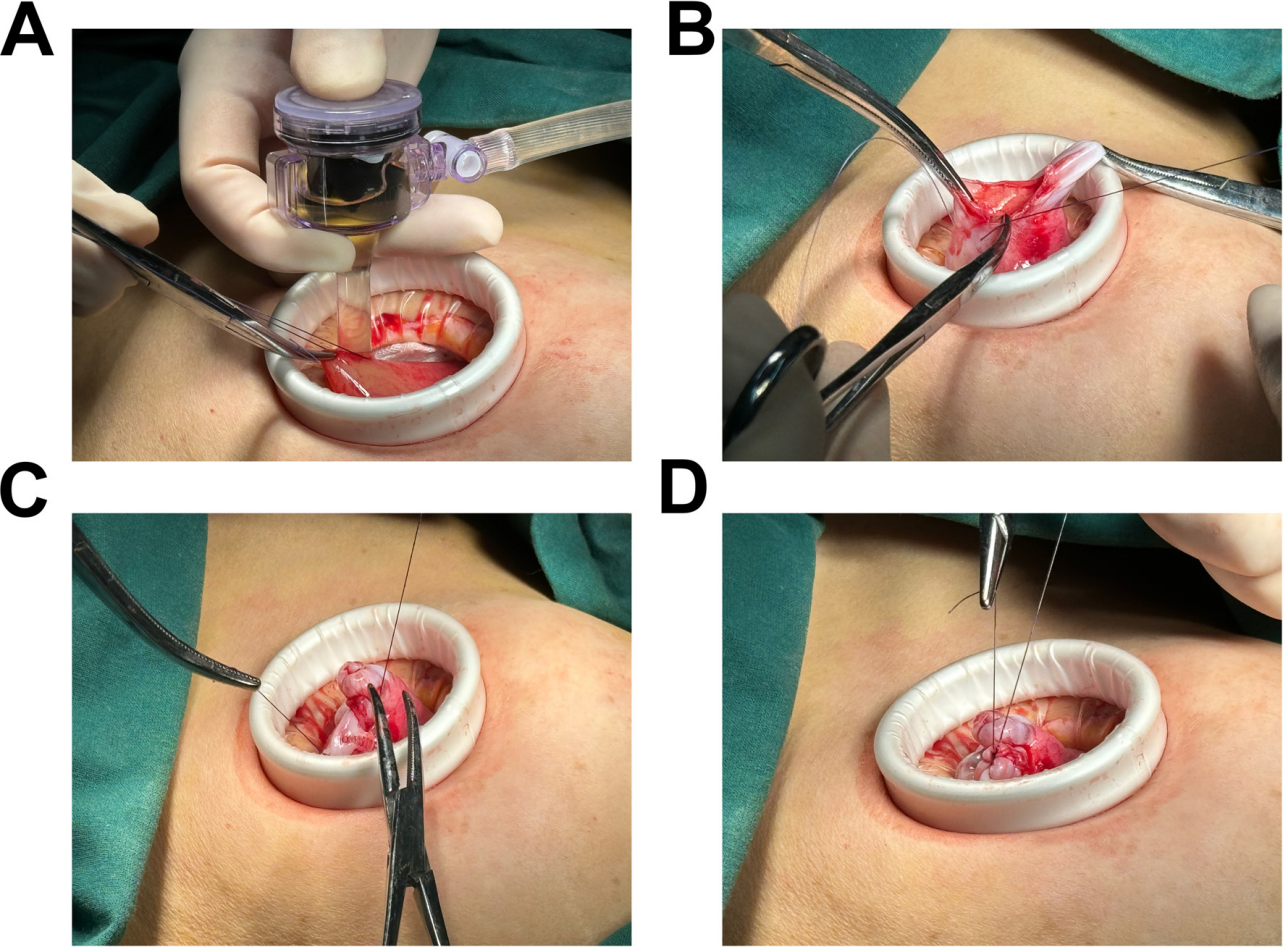
Illustration of the connection between the negative pressure suction device and the 5mm abdominal laparoscopic trocar’s insufflation port. **(A)** The controlled aspiration of cystic fluid was achieved through the precise insertion of a puncture needle, effectively minimizing the risk of fluid leakage and iatrogenic dissemination. Approximately 5000 mL of cystic fluid was aspirated in a slow, methodical manner to ensure safety and efficacy. **(B-D)** Depiction of the final puncture site closure, with suturing performed after the complete aspiration of the cystic fluid.

**Figure 4:**
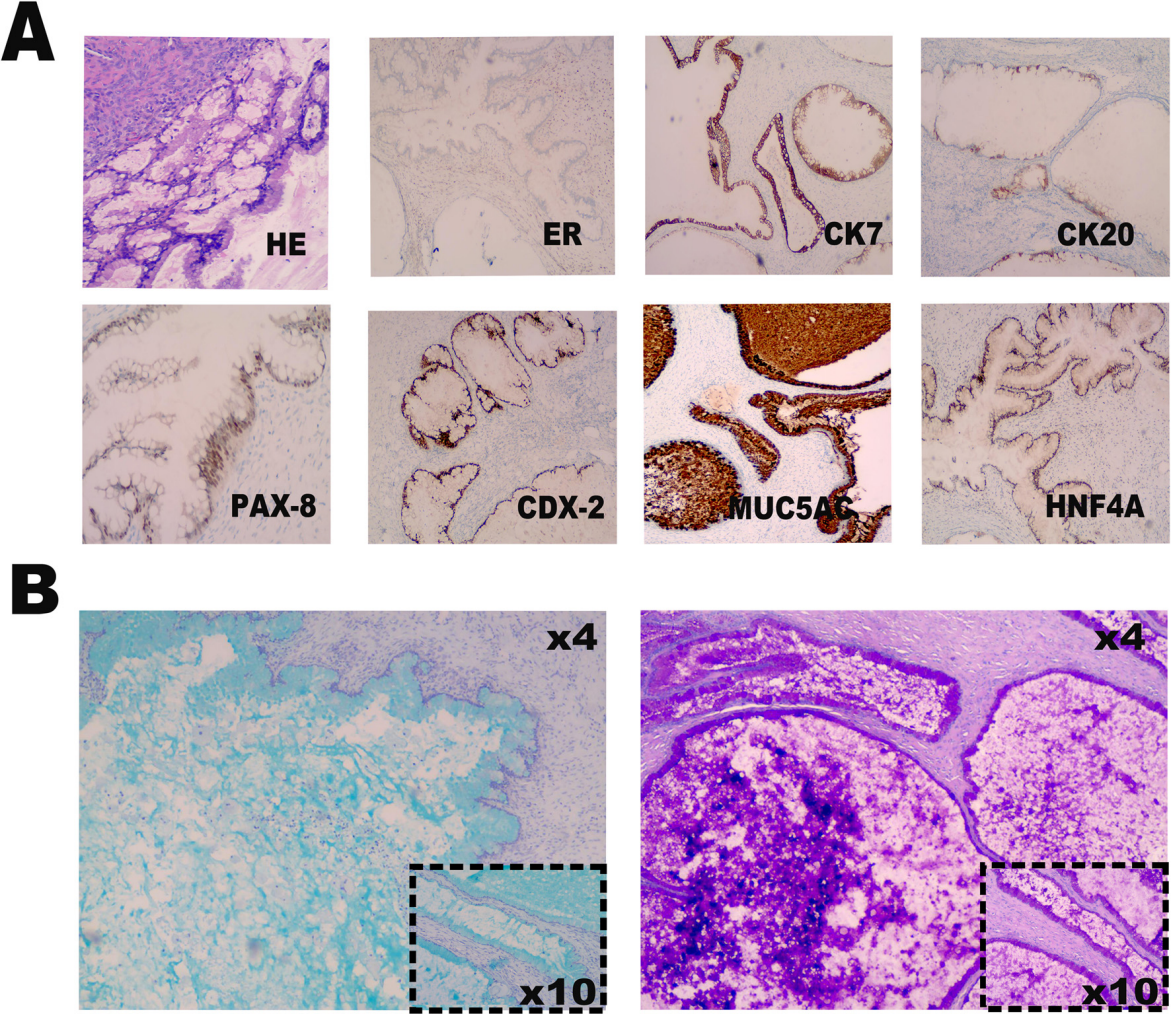
Immunohistochemical and special staining analysis. **(A)** The *he*matoxylin-eosin staining and immunohistochemical marker profile reveals positive expression of CK7, partial positivity for CK20, and strong expression of PAX-8, CDX-2, MUC5AC, and HNF4a. Additionally, ER is negative, and Ki67 demonstrates a proliferation index of 1%. **(B)** Special staining techniques, including Alcian Blue (AB) and Periodic Acid-Schiff (PAS) stains, confirm the presence of mucin positivity.

Laparoscopic examination revealed a large cystic mass with high tension, smooth surface, and clear fluid (**Figures 1C, D**). Due to limited visibility, the source of the mass—either from the pelvic region or retroperitoneum—was unclear. To improve the operative field, we elevated the mass through an umbilical incision and sutured the cyst wall with a purse-string technique to prevent fluid leakage and spillage risks during the procedure (**Figure 2**). As shown in **Figure 3A**, the negative pressure suction device was attached to the 5mm laparoscopic trocar’s insufflation port. The puncture needle was inserted to puncture the cyst wall for controlled aspiration, ensuring stable vital signs and minimizing the risk of fluid leakage. Approximately 5000 mL of cystic fluid was slowly aspirated before carefully suturing the puncture site (**Figures 3B–D**). During laparoscopic re-examination, dense adhesions involving the bowel and omentum were observed in a patient with a history of abdominal hysterectomy for fibroids. A large ovarian cyst had extended into the posterior mesentery, resembling a retroperitoneal tumor. Our surgical team carefully separated the adhesions and excised both adnexa for pathological evaluation. Intraoperative frozen section confirmed a mucinous tumor of the ovary. After obtaining informed consent, the appendix was also removed for further examination. The 2.5-hour surgery was successfully completed without complications, with approximately 100 mL of intraoperative blood loss. The postoperative pathology report confirmed the diagnosis of an ovarian seromucinous cystadenoma. As shown in **Figure 4A**, immunohistochemical analysis revealed the following marker profile: CK7 (+), CK20 (partial +), PAX-8 (+), ER (-), CDX-2 (+), MUC5AC (+), HNF4a (+), and Ki67 (+) at 1%. Special staining techniques demonstrated mucin positivity, as evidenced by both Alcian Blue (AB) and Periodic Acid-Schiff (PAS) stains (**Figure 4B**). The patient’s postoperative recovery was uneventful. Discharge occurred on the third postoperative day without complications. At the 1, 3 and 6-month postoperative follow-ups, the patient recovered well with no abnormalities detected on physical examination, tumor marker testing, or imaging studies.

## Discussion

Ovarian seromucinous tumors are a rare and distinct subtype of ovarian epithelial neoplasms, characterized by the unique histologic presence of both serous and mucinous epithelial components (1–7). Ovarian seromucinous tumors, described by Shappell HW et al. in 2002, are diagnostically challenging due to the complex pathology. In their study of 54 cases, the tumors were found to feature papillary structures with a mix of ciliated serous and cervical mucinous epithelial cells. Researchers emphasized that the diagnosis depends on a detailed assessment of morphology, particularly in differentiating epithelial cells and glandular architecture (2). In 2014, *World Health Organization (WHO)* conducted a substantial revision of the classification of ovarian tumors, leading to the reclassification under the category of seromucinous tumors. This revision highlights the distinctive histological features and clinical signs of seromucinous tumors to enhance diagnostic methods (6). Robert J. Kurman et al. pointed out that while WHO revised classification enhances the understanding of ovarian tumors, it has limitations, particularly concerning seromucinous tumors. These tumors have poorly defined characteristics due to the heterogeneous epithelial types and often show estrogen and progesterone receptor expression, weak WT1 expression, and negativity for CK20 and CDX2, indicating a Müllerian tube immunophenotype. Researchers have also uncovered that occasional co-occurrence of endometriosis may obscure clinical presentations, resulting in interpretations that closely resemble endometrioid tumors, as opposed to the more conventional serous or intestinal-type mucinous tumors. Moreover, a considerable proportion of these tumors demonstrate a loss of ARID1A expression, underscoring their intricate molecular characteristics (8, 9). Subsequent investigations have uncovered that the mutation is found in about 50% of endometrioid and clear cell tumors, but is rare in serous or intestinal-type mucinous tumors. Researchers have suggested reclassifying these tumors as mixed Müllerian tumors due to the distinct clinical, pathological, and molecular features (9–11). In summary, ovarian seromucinous tumors are diagnostically challenging due to the similarity to other neoplasms. Consequently, it is imperative to investigate the unique characteristics to enhance diagnostic methodologies and refine treatment strategies.

Ovarian seromucinous tumors are a rare pathological entity with a relatively low incidence, primarily affecting women during the reproductive years and postmenopausal period (6–12). Our case report presents a 65-year-old postmenopausal female patient, whose clinical presentation is characterized by nonspecific symptoms, predominantly lower abdominal pain and distension. These findings are in alignment with those described in the relevant literature (6–9). The patient presented with abdominal distension, left-sided discomfort, and intermittent morning pain, raising suspicion for an ovarian mass. Imaging revealed a large cystic lesion with solid components that enhanced on contrast-enhanced CT. This case highlighted the diagnostic challenges of ovarian seromucinous tumors, especially when irregularly shaped or in the pelvic region, complicating localization. The mass, measuring 22.8 cm × 12.4 cm × 30 cm, resembled a retroperitoneal tumor due to adhesions and its large size. Several retrospective analyses have demonstrated ovarian seromucinous tumors range from 1.8 cm to 18 cm, with average sizes reported between 9.3 cm and 12 cm (4–14). The ovarian mass we reported has a diameter of approximately 30 centimeters, which is a rare occurrence. Notably, researches have shown that when tumor size exceeds 10 cm, the clinical presentation and diagnosis become markedly more intricate, owing to the heightened risk of metastasis and accompanying complications. Larger tumors are often linked to symptoms such as abdominal distension, pain, and bowel obstruction. As such, the early detection and accurate assessment of tumor size are paramount in evaluating both the feasibility and potential risks associated with surgical resection (6, 7). These findings indicate that the biological behavior of the tumor may be more intricate than previously understood, potentially influenced by factors such as tumor cell differentiation, proliferation rate, and metastatic potential.

The early detection and appropriate management of conditions are essential for optimizing patient outcomes. At present, there is no standardized surgical treatment method. Therefore, management strategies are adjusted based on the patient’s situation, considering variables such as tumor size, anatomical location, and the degree of interaction with adjacent structures (7). In terms of surgical approach, for large or complex ovarian masses, open surgery has traditionally been the preferred option. This methodology not only guarantees superior visualization and access to the tumor, enabling comprehensive excision, but also offers the advantage of facilitating an in-depth assessment of the tumor’s characteristics during the procedure (15). However, with the advancement of surgical instrument technology and the refinement of surgical skills, the innovation of laparoscopic techniques has markedly transformed the surgical landscape, establishing minimally invasive surgery as an increasingly preferred option for the patients. Laparoscopic surgery offers numerous advantages, including reduced trauma, expedited recovery, and diminished postoperative pain, thereby assuming an increasingly pivotal role across a variety of surgical procedures. In comparison to traditional open surgery, laparoscopic approaches facilitate the execution of complex operations through smaller, more precise incisions, resulting in a significant reduction in the incidence of postoperative complications. This evolution represents a substantial advancement in surgical methodologies and fuels the ongoing progress of medical technology (16–18). Single-port laparoscopic surgery (SPLS) represents a significant advancement in the field of gynecological surgery. In comparison to traditional multi-port laparoscopy and open surgery, SPLS offers a notable reduction in invasiveness. The benefits of SPLS are manifold, including minimal scarring, expedited recovery, and diminished postoperative pain, all of which contribute to enhanced patient outcomes and an improved surgical experience. As surgical instruments advance, characterized by innovations such as the development of flexible tools and incision protection devices, and alongside the continuous enhancement of surgeons’ skills, SPLS has increasingly established itself as a fundamental component of contemporary surgical practice (19, 20). SPLS is typically executed through a small incision at the umbilicus, which may be slightly expanded if necessary. This innovative approach not only significantly enhances the surgical view but also minimizes the risk of substantial cystic fluid leakage. Consequently, SPLS showcases distinct advantages in the management of large ovarian masses, particularly in terms of specimen retrieval and mitigating complications associated with trocar placement. As a result, SPLS is garnering increasing recognition and support within the medical community, establishing itself as a progressively valued surgical technique (20). In this SPLS surgery, we employed a suture ring technique to effectively seal a tense, clear fluid-filled cyst, thereby preventing leakage and minimizing the potential dissemination of malignant cells. Controlled suction was utilized to aspirate the fluid, thereby mitigating the surgical risks and complications frequently associated with the management of large ovarian cysts. The presence of abdominal adhesions posed a significant challenge in the surgical management of the patient, particularly following a previous hysterectomy, which complicated tumor identification. This necessitated meticulous dissection to carefully separate the adhesions, thereby underscoring the imperative for precise surgical techniques. The tumor, along with both adnexa, was successfully excised for pathological evaluation, and an appendectomy was additionally performed. While the patient experienced a smooth postoperative recovery, long-term follow-up remains critical due to the potential recurrence of ovarian seromucinous tumors, particularly those exhibiting borderline or invasive characteristics. The role of laparoscopy in the management of these tumors is continually evolving, highlighting the necessity for further research to establish standardized treatment protocols for complex cases.

## Conclusion

Ovarian seromucinous tumors are rare and complex entities, often presenting as large masses that pose significant diagnostic challenges. The application of minimally invasive surgical techniques, such as SPLS surgery, can reduce tissue damage, expedite postoperative recovery, and minimize complication risks. However, achieving successful treatment outcomes requires precise preoperative evaluation, meticulous surgical technique, and comprehensive pathological assessment. Given the limited clinical data available, continuous postoperative surveillance is essential for the early detection of recurrence or malignant transformation.

## Data Availability

The original contributions presented in the study are included in the article/Supplementary Material. Further inquiries can be directed to the corresponding authors.
